# A Pilot Randomized, Controlled Trial of Nighttime Peanut Butter Supplementation in Firefighters: Blood Pressure and Body Composition Outcomes

**DOI:** 10.3390/diseases14040135

**Published:** 2026-04-08

**Authors:** Austin A. Kohler, David H. Shuler, Leke O. Adeleye, Andrew R. Moore, Nicole Peritore, A. Maleah Winkler

**Affiliations:** Department of Kinesiology, Augusta University, 3109 Wrightsboro Road, Augusta, GA 30909, USA; akohler@augusta.edu (A.A.K.); shulerdavid@gmail.com (D.H.S.); adeleye_leke@yahoo.com (L.O.A.); andmoore@augusta.edu (A.R.M.); nperitore@augusta.edu (N.P.)

**Keywords:** systolic blood pressure, diastolic blood pressure, body fat percentage, fat mass, monounsaturated fatty acids, functional foods

## Abstract

**Background/Objectives**: Dietary approaches to combating risk factors for cardiovascular disease are valuable, especially for individuals in high-stress occupations like first responders. The purpose of this pilot randomized control trial was to determine the effect of regular peanut butter (PB) supplementation on blood pressure and primary measures of body composition (body fat %, fat mass, and lean mass) in firefighters. **Methods**: Full-time firefighters (N = 40; 1 woman) were randomly assigned to a control group or a peanut butter group for 7 weeks. Participants in the peanut butter group consumed one serving of peanut butter before bed at least 5 days per week for the intervention period. Participants in the control group continued with their usual diet. Indices of body composition and blood pressure were collected before and after the intervention period and compared using mixed-factorial ANOVAs (α = 0.05). **Results**: No interaction effects between group and time were observed for blood pressure variables (*p* = 0.619–0.650). Similarly, the changes among the PB group over time in percent body fat (Δ = −0.53 ± 1.74%), fat mass (Δ = −0.73 ± 2.21 kg), and lean body mass (Δ = 0.04 ± 1.65 kg) were not significantly different than the changes over time in the control group (*p* ≥ 0.067 for all). **Conclusions**: Seven-week PB supplementation did not affect male firefighter body composition or blood pressure; however, future studies should investigate longer durations with sophisticated dietary recall methods. ClinicalTrials.gov Identifier: NCT06364202.

## 1. Introduction

Peanuts are inexpensive, palatable, and may exert many beneficial health effects on blood pressure and body composition due to their bioactive compounds, which include antihypertensive and vasodilatory properties [1,2,3]. They are also high in fiber, protein, vitamins, and minerals and have been shown to reduce circulating lipid levels and the risk of CHD when consumed as part of the diet [4,5]. Peanuts may positively influence body composition by reducing weight, body mass index (BMI), and waist circumference [6,7]. Research has shown that the high protein and fiber content of peanuts may improve body composition through appetite regulation, increased satiety, and increased energy expenditure [1,8,9]. Peanut butter is a convenient alternative to whole peanut consumption and has minimal nutrient loss during processing [10]. However, questions remain regarding the quantity, timing, and duration of consumption that would be required to elicit meaningful changes in blood pressure and body composition.

Many aspects of the firefighting occupation place significant strain on the cardiovascular system, including shift work and resulting sleep deprivation, chronic sympathetic nervous system activation from alarms and emergency response, performance of intense physiological tasks in extreme and hazardous environments, dehydration, and psychological stress from experiencing and reliving unsuccessful rescues [11,12,13,14]. In addition, poor nutrition emanating from inconsistent eating times and limited food options, as well as long sedentary periods waiting for a call, may further impair the health of the cardiovascular system [11,15]. These occupational demands and situations that challenge the cardiovascular system often promote elevated blood pressure and obesity, which are major risk factors for cardiovascular disease (CVD) [16,17,18].

CVD accounts for almost half of the deaths that have occurred in the US fire service, thus making it the leading cause of death among firefighters while on duty [19,20]. Coronary heart disease (CHD) is the most prevalent form of CVD in firefighters, and 45% of the on-duty CVD-related fatalities have originated from CHD [19]. A large cohort of U.S. firefighters demonstrated high prevalence of cardiometabolic risk factors, including high blood pressure (81% men and 58% women), low HDL cholesterol (36% men and 45% women), and metabolic syndrome (18% men and 10% women) [13]. Hypertension is prevalent in firefighters, with approximately 50% of firefighters classified as prehypertensive and 20–30% as hypertensive [11,15]. Although blood pressure control is imperative to reducing the risk of CVD [21], Soteriades et al. reported that 75% of hypertensive firefighters did not take measures to monitor and control their condition [22]. Additionally, uncontrolled hypertension has been linked to CHD incidents and fatalities, as well as heart disease-based disability and early retirement from active duty [15,23].

Evidence of the increased cardiovascular risk was examined in a large 13-year follow-up study by Noh et al., which reported that while hypertension increased the risk of major adverse cardiovascular events (MACEs) in both firefighters and controls, firefighters exhibited a significantly greater risk overall, and elevated blood pressure was associated with increased MACEs only in the firefighter group [24]. Furthermore, firefighters display a concerning rate of obesity, which is influenced by shift-work schedules, inconsistent mealtimes, poor firehouse nutrition, and/or lack of physical activity due to waiting for calls [16,17,25,26]. Additionally, the Firefighter Heart Disease Prevention study corroborated findings and found that over one-third of asymptomatic active-duty firefighters in Georgia had subclinical coronary calcification, which increases the risk for a cardiovascular event [14]. Because excess adiposity and elevated blood pressure frequently coexist in firefighters and jointly increase cardiovascular risk, interventions that are effective in reducing cardiovascular risk and feasible to incorporate within the firefighting occupational setting are of particular interest. Applicable interventions to address these health concerns for firefighters are needed; however, adherence to traditional dietary strategies remains challenging. Kay et al. revealed that although firefighters understood that proper dietary choices could reduce their risk for CVD, 37% reported that they had no intention of changing their diet [27]. Furthermore, the nature of shift work and unpredictable emergency calls is likely to hinder a firefighter’s capacity for meal planning and increase the probability of stopping for fast food [18,28]. Shift work has been shown to negatively affect both the timing and quality of dietary intake [25].

Research has demonstrated the health benefits of peanut consumption, and peanut butter could be a quick, easy, and nutritious food option for firefighters who have a unique schedule and workplace activity. However, limited research has examined the effects of peanut butter consumption when timed to align with firefighters’ occupational schedules, particularly with respect to their blood pressure and body composition outcomes. Therefore, the purpose of this study was to determine whether consumption of peanuts in the form of peanut butter before bedtime for seven weeks altered blood pressure (systolic and diastolic) or body composition (body fat percentage (%), fat mass, and lean body mass) in full-time firefighters. We hypothesized that: (H1) consuming peanut butter for 7 weeks would reduce systolic and diastolic blood pressure in firefighters and (H2) consuming peanut butter for 7 weeks would reduce body fat % and fat mass in firefighters without reducing lean body mass. This study was designed as a feasibility-focused pilot trial to determine whether an affordable, nighttime dietary intervention could be implemented in firefighters and produce early cardiometabolic changes under real-world conditions.

## 2. Materials and Methods

### 2.1. Experimental Design

To determine if consuming peanut butter prior to bedtime impacts blood pressure and/or body composition, an 8-week parallel-arm randomized controlled trial was conducted, comprising 40 full-time firefighters. On the first day of the 8-week study (1 week baseline, 7 weeks intervention), demographic information and baseline physiological data, including blood pressure and body composition, were collected, and an activity monitor in the form of a wristwatch was provided. The participants were briefed on the activity monitor and instructed to wear the device for the first full week of the study to collect daily step counts as the baseline measure. Following the baseline week, participants were randomized by a blinded statistician into either a peanut butter (PB) group or a control group and remained in their designated group for the following 7 weeks. Throughout the 7-week intervention period, participants continued to wear the activity monitor daily, regardless of whether they were on shift, and completed subjective questionnaires twice a day. Data from these secondary measurements were collected to evaluate physical activity level and subjective sensations related to appetite for any changes in blood pressure or body composition. At the end of the 7-week period, blood pressure and body composition data were collected again.

A 7-week intervention period was selected as a practical and feasible period for this population of full-time firefighters who work shifts of 24 h on and 72 h off, which often cause scheduling challenges. These work patterns complicate the implementation of longer, standardized dietary studies that require high adherence. As a pilot randomized controlled trial, the 7-week duration enabled us to evaluate feasibility, participant adherence, and initial physiological responses prior to designing a longer study of 12 to 24 weeks, which has been used in other peanut-related nutrition research.

Participants received a copy of the informed consent via email to review prior to starting the study and then signed the informed consent prior to baseline data collection. This study was approved by the University’s Institutional Review Board (IRBnet ID# 1928368), and all procedures performed followed institutional guidelines. This study was registered as a clinical trial (ClinicalTrials.gov Identifier: NCT06364202), and raw data were collected from October 2022 until August 2023 in the USA, Georgia.

### 2.2. Participants

**Figure 1:**
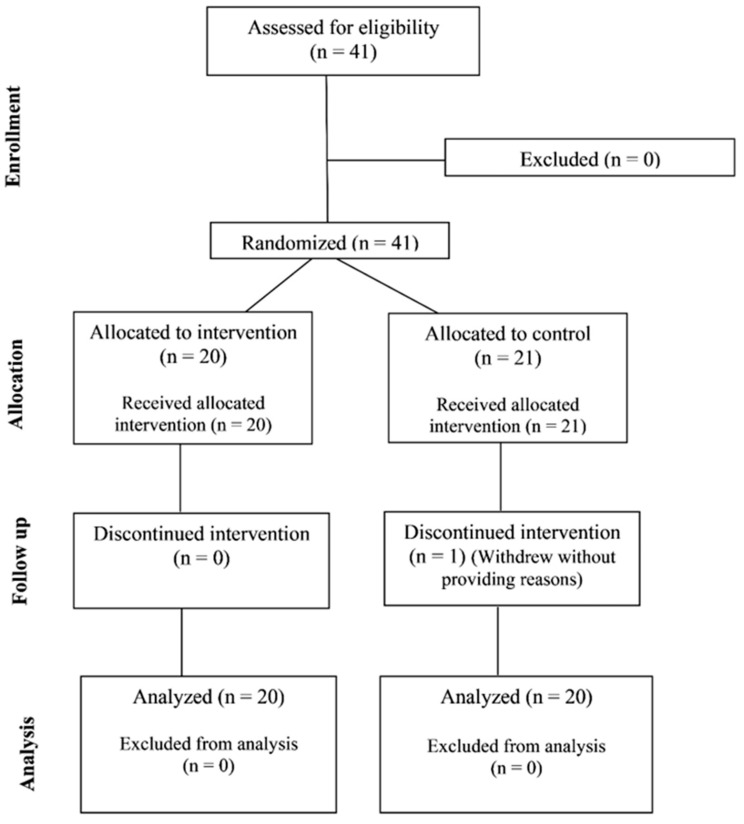
Flow diagram demonstrating the number of participants recruited, enrolled, and analyzed.

To participate in this study, participants needed to meet the following criteria: at least 18 years old, currently employed as a full-time firefighter, and without a known peanut allergy. No other health criteria were used, so the results would apply broadly to firefighters, who often face issues such as high blood pressure, being overweight or obese, or cardiometabolic risk. Having a history of hypertension or other chronic diseases did not prevent participation. People taking medications such as antihypertensives or antidiabetic drugs could join as long as they reported any changes in their medication during the study. No medical or prescription changes were disclosed throughout the study. A power analysis was computed (G*Power version 3.1, Düsseldorf, Germany) to estimate the required sample size needed to observe a within-between interaction effect for an ANOVA. The estimated effect size was set conservatively at f = 0.25 (small-moderate effect size) due to no reported effect sizes for published studies using a similar intervention. With two groups, at two timepoints, an alpha level of 0.05 and a power of 0.80, a sample size of 34 participants was estimated to be required. Additional participants were recruited to take part to offset potential dropouts from the study. Forty-one full-time firefighters (40 males, 1 female) from the same fire department located in the southeastern part of the United States, who worked shifts of 24 h on and 72 h off, began the study. However, data from only 40 participants were formally analyzed following the dropout of one participant, as shown in Figure 1. Table 1 includes the participant characteristics for the 40 firefighters whose data were analyzed, along with the *t*-test results of baseline comparison between each group of participants.

### 2.3. Protocol

The study consisted of a 1-week baseline period and a 7-week intervention period for a total participation time of 8 weeks. Two data collection visits occurred; the first visit occurred on the first day of the baseline week, and the second visit occurred immediately after the 7-week intervention period. Although prior medical history and medication use were not exclusionary, participants were instructed to maintain their usual medication routines throughout the study. Because no medication changes were disclosed, the potential confounding influence of altered medication regimens during the intervention period was minimized.

To ensure the accuracy of blood pressure and bioelectrical impedance analysis (BIA) measurements, participants were instructed to abstain from eating and exercising for 8 h and from consuming caffeine and nicotine for 12 h prior to the baseline and post-intervention data collection visits. Hydration status was not directly assessed prior to measurements; however, these standardized pre-assessment instructions were intended to minimize acute fluctuations in hydration status and physiological factors known to influence BIA-derived estimates. Participants were encouraged to hydrate regularly with water prior to the laboratory visits.

Both visits started with resting blood pressure measurements. After resting in a seated position for 10 min, blood pressure was measured twice with an automated blood pressure cuff (Omron 10 Series Blood Pressure Monitor Model BP785N; Omron Healthcare, Inc., 1925 West Field Court, Lake Forest, IL, USA). Age and height were self-reported by the participants. Body composition variables, including weight, body fat mass content and percentage, and lean mass content, were measured via a BIA stand-on scale (Tanita DC-430U Dual Frequency Total Body Composition Analyzer, Arlington Heights, IL, USA). Similar foot-to-foot models of BIA devices, from this manufacturer and others, have high test–retest reliability in adults (r ≥ 0.991) [29] and have been used in other studies assessing body composition in firefighters [30]. The same device and testing procedures were used for all participants at both time points to ensure consistency of repeated measurements. BMI was determined and recorded as a secondary outcome variable because it is a measure that is freely and readily available to personnel at all fire stations.

At the end of the first visit, an actigraphy wristwatch (ActiGraph wGT3X-BT, Pensacola, FL, USA) was strapped onto the participants’ wrist to wear throughout the study to measure step counts during the baseline week and throughout the 7-week intervention period. Participants were given a charger to charge the monitor if it started blinking red, indicating low power, or an investigator switched the monitor for a fully charged monitor. Participants were sent a visual analog scale (VAS) via a Qualtrics survey in the morning and evening of each day throughout the baseline week and 7-week intervention period, which assessed subjective hunger, satiety, and energy. Each element was reviewed on a 1–10 Likert scale, and respondents reported on their current status. The hunger scale ranged from “1”, indicating “I’m stuffed, can’t eat anymore”, to “10”, indicating “So hungry, it hurts.” The satiety scale ranged from “1”, indicating “I don’t feel full at all”, to “10”, indicating “I feel very full.” The energy scale ranged from “1”, indicating “I feel very tired”, to “10”, indicating “I feel more energized.”

**Figure 2:**
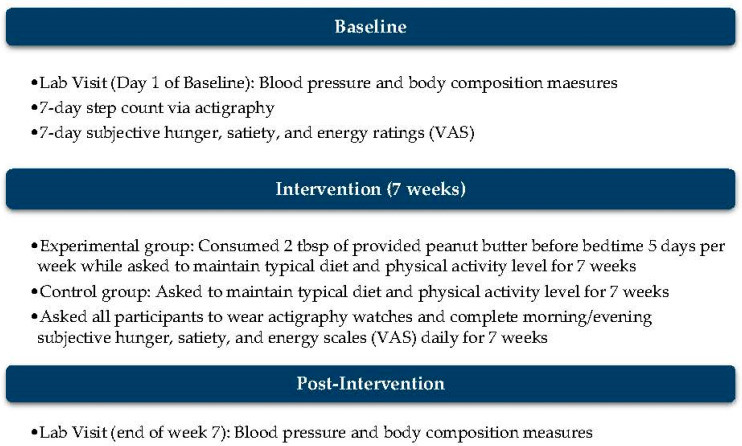
Outline of protocol throughout the 8-week period, which includes 1 week for baseline measures and 7 weeks for the intervention.

Participants were randomized into a PB or control group after the baseline week. For the 7-week intervention period, both groups were asked to maintain their typical diet and physical activity level with the addition of ending each day’s eating period 2 h prior to bedtime for 5 days per week. The PB group was also asked to consume one serving of peanut butter (Smooth Operator, Peanut Butter & Co, New York, NY, USA) 2 h prior to bedtime, 5 days per week, which totaled 35 days throughout the 49-day intervention period. The methods are outlined in Figure 2 for visualization. Objective measures of total energy or macronutrient intake, medication use, or compliance with the fasting window were not collected. All dietary recommendations were self-directed by participants.

The peanut butter was provided in 32-g single-serving packages, which were approximately two tablespoons of peanut butter. The 32 g of peanut butter included peanuts, cane sugar, palm oil, and salt and equated to 190 calories, 15 g of total fat, 8 g of dietary carbohydrate, 2 g of added sugar, and 7 g of protein.

To improve adherence, a reminder text message was sent daily, and peanut butter packages were provided every two weeks. Participants were asked if they had consumed the peanut butter prior to providing the next allotment.

### 2.4. Statistical Analysis

All analyses were completed with SPSS, version 29 (IBM, Armonk, NY, USA) using an alpha level of 0.05. Data in each group and at each time point were screened for outliers (>3.29 SD units from the group mean). Data was also screened for normality using the Shapiro–Wilk test. Violations of the assumption of normality are reported, but no transformations were made to correct for any violations because ANOVA is robust to violations of normality [31]. The assumption of homogeneity of variances was assessed using Levene’s test of homogeneity of variance (α = 0.05). The assumption of homogeneity of covariance was assessed using Box’s test of equality of covariance matrices (α = 0.001).

A series of mixed 2 × 2 factorial ANOVAs was used to analyze the effects of intervention group (group) and intervention time (time) on the main variables of interest. The between-subjects factor was group and consisted of two levels (control or PB). The within-subjects (i.e., repeated measures) factor was time, which consisted of two levels (PRE and POST). In addition to statistically evaluating the main effects of group and time, each of the ANOVAs also statistically evaluated the interaction of the factors group and time on the primary dependent variables, systolic blood pressure (SBP), diastolic blood pressure (DBP), body fat %, fat mass, and lean body mass, and the secondary variable, BMI. In the event of significant findings, Bonferroni-adjusted post hoc tests would be used as appropriate.

VAS data were intended to be analyzed with mixed factorial ANOVAs due to having more than two timepoints. However, substantial amounts of missing data prevented the use of ANOVAs for analysis of changes in the VAS variables. Therefore, the variables hunger, satiety, and energy were analyzed using separate linear mixed-effects model analyses. Individual subjects were specified as a correlated random effect to limit pseudo-replication of results. Averages for each week in the morning (AM) and evening (PM) were computed and designated as the repeated-measures variables Week with 8 levels (baseline and weeks 2–8) and time of day (TOD) with two levels (AM and PM). The fixed factors were Week, TOD, and Group (control or PB). A compound symmetry covariance structure was selected for the repeated-measures factors in all mixed-effects models. Tests of fixed effects were generated along with Bonferroni-adjusted post hoc tests to maintain an alpha level of 0.05. Effect size is reported as partial eta squared (*η*^2^) for ANOVA results. Effect sizes are interpreted for *η*^2^ as small (*η*^2^ = 0.01), medium (*η*^2^ = 0.06), and large (*η*^2^ = 0.14) as described by Cohen [32].

The number of steps taken per day was compared between the control and PB groups at each week of the study using a linear mixed-effects model analysis, as with each of the VAS variables. This was done to compare the average daily step count in each group over the entirety of the intervention period. Individual subjects were specified as a correlated random effect, Week was the repeated measures fixed factor, and Group was the between-participants fixed factor.

Two participants had values for several variables that were considered outliers, notably body fat % values > 43% and BMI values > 55. While these levels of obesity may not be representative of the majority of firefighters, these participants were still part of the population of interest. As such, we completed all analyses with and without these two participants who exhibited severe obesity. No material difference was found in the statistical outcome for any of the analyses, so the presented results reflect the data from all participants who completed the study protocol. One participant did not complete data collection for the POST time point and dropped out due to personal reasons/scheduling conflicts. Therefore, although 41 participants started the study, only the data from 40 of these participants were formally analyzed. Some other participants were missing data for certain variables (i.e., systolic blood pressure) and were removed from these respective analyses via pairwise deletion. There were participants with missing accelerometer data (device malfunction, improper use, forgetting to put on the device, etc.), which can be seen with the different sample sizes within the analyses.

## 3. Results

There were several sets of scores for body composition and blood pressure variables, which violated the assumption of normality. In the control group, BMI (*p* = 0.004), fat mass (*p* < 0.001), and lean mass (*p* = 0.018) were non-normal at the PRE time point, and DBP (*p* = 0.020), BMI (*p* = 0.001), fat mass (*p* < 0.001), and lean mass (*p* < 0.038) were non-normal at the POST time point. In the peanut butter group, BMI (*p* < 0.001) and fat mass (*p* < 0.001) were non-normal at the PRE time point, and BMI (*p* < 0.001) and fat mass (*p* < 0.001) were non-normal at the POST time point. No violations of the assumptions of homogeneity of variance or homogeneity of covariance were detected. Normality violations were also detected among the following sets of VAS scores: hunger and satiety in the Control group, in week 7, PM; hunger in the PB group, in Week 1, AM; energy in the PB group, in Week 6, AM; and energy in the PB group, in week 6, PM. The datasets analyzed for this study can be found in the corresponding project page in the Open Science Framework [https://osf.io/8xs5h/overview (accessed on 4 April 2026)].

### 3.1. Blood Pressure and Body Composition Variables

Complete descriptive statistical results for blood pressure and body composition variables are located in Table 2. Inferential statistical results are seen in Table 3. Additionally, differences in baseline scores between groups were examined. No significant differences for any blood pressure or body composition variables were detected. There was no significant interaction effect between group and time on SBP, DBP, body fat %, fat mass, lean mass, or BMI. No significant main effects of group or time were detected for any of these variables.

### 3.2. VAS

Complete descriptive statistical results for VAS analyses and inferential statistical results can be found in the corresponding project page in the Open Science Framework [https://osf.io/8xs5h/overview (accessed on 4 April 2026)]. There were no significant interaction effects between any of the variables, group, week, or TOD on hunger, satiety, or energy.

There was a main effect of TOD on hunger [F(1, 518.56) = 141.89, *p* < 0.001], which was higher in AM than in PM (mean difference = 1.011, CI95 = 0.844, 1.177). There was a main effect of TOD on satiety [F(1, 518.51) = 198.24, *p* < 0.001], which was higher in PM than AM (mean difference = 1.252, CI95 = 1.077, 1.427). There was also a main effect of week on satiety [F(7, 519.21) = 2.12, *p* = 0.040], but the difference was deemed non-significant following Bonferroni adjustment for multiple comparisons. There was a main effect of TOD on energy [F(1, 518.02) = 60.86, *p* < 0.001], which was higher in AM than PM (mean difference = 0.591, CI95 = 0.442, 0.740). All other main effects were not significant. Complete descriptive and inferential statistical results are available at the data repository linked at the end of the manuscript.

### 3.3. Daily Step Counts

There was no significant difference in daily step count between the Control [M = 11,295.5, 95%CI = (9864.0, 12,727.0)] and PB [M = 11,375.5; 95%CI = (9955.8, 12,727.0)] groups over the course of the eight-week intervention period [F(1, 43.34) = 0.004, *p* = 0.951, Mdiff = −62.0, 95%CI = (−2065.4, 1941.5)]. Weekly averages for each group are provided in Table 4, along with post hoc test results of between-group comparisons at each time point.

## 4. Discussion

After eating peanut butter two hours before bed for seven weeks, the blood pressure and body composition of full-time firefighters were assessed in this pilot randomized controlled study. There were no statistically significant group-by-time effects for any of the primary or secondary outcomes, indicating that the intervention did not produce discernible cardiometabolic changes during this time frame.

According to baseline measures, both the control and PB groups had an average elevated blood pressure, defined as SBP of 120–139 mmHg and/or DBP of 80–89 mmHg. Specifically, the control group averaged a systolic/diastolic blood pressure of 123.5/77.6 mmHg while the PB group averaged 130.0/81.0 mmHg at baseline. Both groups met the systolic blood pressure criteria and remained in the elevated category. Nouran et al. demonstrated similar blood pressure outcomes in hypercholesterolaemic men who consumed peanuts for 4 weeks compared to a control group that did not [7]. Blood pressure did not significantly change with peanut consumption; however, the control group averaged normal blood pressure at baseline, and the peanut group averaged a low-end elevated DBP of 80 mmHg with a normal SBP. The study demonstrated reduced estimates of coronary heart disease risk with peanut consumption based on both SBP and DBP results.

On the other hand, Petersen et al. demonstrated a reduction in SBP compared to a control group in adults at risk of type 2 diabetes when they consumed 35 g of peanuts before two main meals for 6 months [8]. Similar to our study, the participants averaged an elevated blood pressure of 128/81 mmHg. Jones et al. demonstrated the importance of assessing the effects of peanut consumption on the blood pressure of participants with elevated baseline blood pressure compared to normal blood pressure [9]. Participants in this study were randomized into different flavor peanut groups to assess if salt, sugar, or flavors make a difference compared to consuming unsalted peanuts for 12 weeks. Overall, there was a significant decrease in DBP, with the salted and unsalted peanut groups showing greater decreases than the spicy or honey-roasted peanut groups. In addition, blood pressure between participants who had a normal blood pressure (≤120/80 mmHg) and those with an elevated blood pressure (>120/80 mmHg) at baseline was compared. The group with the elevated blood pressure had significantly greater reductions in DBP than the group with normal blood pressure.

In this study, the baseline BMI for the control and PB groups was similar (30.4 vs. 31.9), resulting in classification of obese (BMI ≥ 30) in both cases. These classifications remained the same at the end of the 8-week period, reflecting the lack of significant changes in BMI, body fat%, and fat mass between the two groups.

Although short-term nutrition and exercise programs have been shown to improve body composition, the lack of significant changes in the present study may reflect the practical challenges firefighters face in sustaining more intensive interventions [33,34,35]. Previous work suggests that even structured dietary approaches, such as the Mediterranean diet, can be difficult for firefighters to maintain over time despite their known cardiometabolic benefits [35]. In this context, the simple and minimally disruptive peanut butter intervention used in the present study may not have been sufficient to produce measurable changes in body composition over seven weeks. Together, these findings demonstrate that the feasibility and consistency of dietary strategies may be as important as their nutritional quality when addressing cardiometabolic health in firefighters. The reason that peanuts and the consumption of peanut butter were selected for this study was due to the health benefits of the food and the ease of access and consumption (i.e., familiar item and easy to eat).

Short-term nutrition interventions lasting 6 to 8 weeks are common in cardiometabolic and bioactive compound research. For instance, an 8-week randomized, placebo-controlled trial of polyphenol-rich grape seed extract measured ambulatory blood pressure in people with pre- or stage I hypertension [36]. Polyphenol-rich juices have also shown measurable reductions in blood pressure within the first 6 weeks of intervention [37]. Studies on peanuts and other nuts often use short durations as well, such as 4-week peanut and peanut butter trials that assess body weight and plasma lipids [38], and several nut consumption trials lasting 4 to 8 weeks were included in meta-analyses [39]. A recent 8-week peanut butter intervention study has also been completed [40]. Together, these studies show that short interventions of 4 to 8 weeks, similar to our 7-week study, are standard in bioactive nutrition research and useful for evaluating feasibility and early physiological outcomes.

Multiple physiological reactions may occur from consuming nutrients derived from peanuts over a shorter time period, like 7 weeks. Foods high in arginine, such as peanuts, may acutely increase the availability of nitric oxide, resulting in brief vasodilation and slight short-term decreases in vascular resistance [3]. Magnesium and potassium can also affect vascular smooth muscle tone and endothelial function within the first weeks of ingestion, which may lead to early improvements in blood pressure. Short-term nut interventions of 4–8 weeks have shown small reductions in blood pressure or circulating lipids, which indicate that early physiological changes are possible but subtle [39]. These mechanisms could lead to slight changes in cardiometabolism over the course of seven weeks; however, our feasibility-focused pilot study did not observe significant cardiometabolic improvements, possibly from the combination of small, expected effect sizes, added sodium in the peanut butter, and real-world dietary variability.

While diet plays a major role in health outcomes, physical activity also contributes. The common recommendation for physical activity is 10,000 steps per day for health improvements and maintenance [41]. To determine if the firefighters were meeting physical activity recommendations and maintaining normal activity levels throughout the study period, daily step counts were measured and averaged per week over 8 weeks. Both groups averaged at least 10,000 steps per day each week and exceeded this amount for all weeks of the study apart from one week for the PB group. The average across the 8-week study was less than 0.01% (62 steps/day) between groups, suggesting that it is unlikely that the small but potentially meaningful improvements in body composition in the PB group were due to extra physical activity and caloric expenditure in that group.

One strength of the study was that participants were from the same general geographical location and therefore subject to similar types and amounts of occupational stress and health disparities. The study was randomized and counterbalanced, although blinding participants to their condition was not possible, preventing self-selection of groups, which limited some sources of confounding variables. We acknowledge that the results are limited in part by a lack of experimental control or monitoring of some dietary and lifestyle aspects of the study participants. However, the results presented are more generalizable to the population of interest because the data were collected under conditions that mirror the complexities of full-time shift-working firefighters. Specifically, participants maintained a typical diet and physical activity regimen and self-implemented the instructions of the PB condition. This decision was made to incorporate an element of ecological validity, since any dietary intervention for firefighters is subject to the occupational demands and priorities associated with this population, and controlling caloric intake over an unpredictable 24-h shift is not feasible. Nonetheless, the absence of dietary intake data may prevent identifying the independent effects of peanut butter supplementation. This study included multiple limitations in the study design and outcome assessments. Regarding physical activity assessment limitations, step count was used to measure physical activity levels in the firefighters, which does not consider the intensity of the activity. Such physical activity details might prove valuable in untangling the reason for body composition improvements in some participants in the PB group, but not in others. Participants were asked to wear the actigraphy wristwatch throughout the intervention; however, due to the watch recharging and/or participants taking the watch off for various periods (i.e., during a shower), some time periods were missing throughout the 8-week period. Regarding physiological assessment limitations, the participants self-reported height from their most current yearly physical, which may add a source of error. Also, hydration status was not directly measured prior to the BIA body composition assessment, which may influence the absolute accuracy of body composition estimates. However, the participants did follow standardized pre-testing instructions for BIA measurements, and the same device was used at both baseline and post-intervention, which likely reduced the impact on within-subject change. Lastly, regarding study design limitations, the participants and investigators were not blinded. However, the assessments were automated, which reduced bias from the investigators. Also, the control group did not receive a placebo; full double blinding was not possible, which may have influenced internal validity. Failure to blind participants and investigators to the intervention may have also affected appetite measures in the intervention group, as it was not placebo-controlled.

The results of this pilot study yielded several recommendations for follow-up investigations into the effectiveness of peanut butter supplementation on health outcomes in this population. Future explorations should assess peanut butter consumption over a longer period of 3 to 6 months, as the 7-week period used in this study may have been insufficient to detect changes in blood pressure or body composition. Participants should include firefighters with elevated blood pressure and those who are categorized as being overweight or obese. Additionally, sophisticated dietary recall methods, such as videotaping meal preparation and consumption or recording food consumption during meals using an automated electronic scale, should be included to determine if energy intake differs between the peanut butter consumers and control groups to identify the effects of peanut butter more accurately on these measures of health. Precise adherence percentages (e.g., ≥80% compliance) could not be determined because adherence was tracked verbally during biweekly peanut butter distribution meetings rather than recording empty packets. Lastly, the peanut butter selected for supplementation was chosen because it came in pre-portioned single-serving packets that ensured each participant consumed the same amount, and its favorable taste ratings were expected to help support consistent intake. However, the peanut butter contained added salt and palm oil, which may have attenuated expected cardioprotective benefits from the bioactive compounds in peanuts; thus, future studies should consider using peanut butter made solely from peanuts to avoid any potential influence of these added ingredients on study outcomes. Taken together, these limitations do not invalidate the results; however, they may have prevented the detection of minor physiological changes. Thus, this study serves as a feasibility pilot providing support for a future larger efficacy trial.

## 5. Conclusions

Nightly peanut butter supplementation for 7 weeks did not reduce blood pressure or body composition in predominantly male firefighters. This pilot clinical trial is limited by short-term intervention duration, lack of assessment of dietary intake, lack of blinding, and lack of female participants and thus has limited generalizability to female firefighters. Nonetheless, data from this pilot clinical trial provide feasibility data to inform a future study of extended duration that will better describe the clinical efficacy of nighttime peanut butter supplementation in full-time firefighters. Future trials should implement longer interventions of 3 to 6 months, as that duration has been used in prior nut and peanut-based dietary studies and may be essential for identifying significant alterations in blood pressure and body composition. Additionally, future studies should assess potential mechanistic pathways, including measures of endothelial function, nitric oxide bioavailability, lipid profiles, and inflammatory markers, to determine the physiological effects of consuming single-ingredient peanut butter formulations. These future study alterations will help determine whether peanut butter is a beneficial and feasible dietary strategy for improving cardiometabolic health in firefighters.

## Figures and Tables

**Table 1:** Anthropomorphic and demographic information for participants. Data are presented as mean values followed by standard deviation values in parentheses for the control group, peanut butter (PB) group, and for both groups combined (All).

	Control (*n* = 20; 0 Women)	PB (*n* = 20; 1 Woman)	All (*N* = 40; 1 Woman)
Age (years)	36.30 (6.60)	33.35 (9.07)	34.83 (8.04)
Height (cm)	178.94 (7.02)	178.31 (7.49)	178.62 (7.17)
Weight (kg)	97.51 (27.39)	101.13 (26.25)	99.32 (26.54)
BMI (kg/m^2^)	30.35 (7.69)	31.89 (8.36)	31.12 (7.97)
Body Fat (%)	23.18 (7.90)	25.96 (10.14)	24.61 (9.11)
Race (*n*)			
White	18	16	34
Black/African American	2	3	5
Asian	0	1	1
Ethnicity (*n*)			
Hispanic	1	1	2
Non-Hispanic	19	19	38

**Table 2:** Descriptive results for blood pressure and body composition variables before (PRE) and after (POST) the intervention period. The mean value is presented, followed by the standard deviation in parentheses. Effect size is reported as Hedge’s correction (*g*).

	Control			PB		
	PRE	POST	*g*	PRE	POST	*g*
SBP (mmHg)	123.5 (10.0)	123.5 (8.3)	0.00	130.00 (14.1)	128.44 (13.0)	−0.13
DBP (mmHg)	77.6 (9.1)	78.7 (10.1)	0.12	81.00 (13.4)	80.90 (12.5)	−0.01
Body fat %	23.2 (7.9)	23.5 (8.2)	0.22	26.0 (10.1)	25.4 (9.8)	−0.29
Fat mass (kg)	24.4 (16.8)	25.1 (18.2)	0.26	28.5 (19.7)	27.7 (19.5)	−0.32
Lean mass (kg)	69.4 (12.2)	69.5 (11.2)	0.06	69.5 (8.7)	69.6 (8.8)	−0.02
BMI (kg/m^2^)	30.4 (7.7)	30.6 (7.8)	0.32	31.9 (8.4)	31.6 (8.2)	−0.26

Notes: PRE = time period before the intervention started; POST = after the intervention period; PB = Peanut Butter group; SBP = systolic blood pressure; DBP = diastolic blood pressure; Body fat % = body fat percentage; BMI = body mass index. Effect size (*g*) is interpreted as small (*g* = 0.2), medium (*g* = 0.5), and large (*g* = 0.8).

**Table 3:** Statistical results for blood pressure and body composition variables for the variables group (Control or Peanut Butter), time (PRE and POST), and their interaction. Effect size is reported as Hedge’s correction (*g*).

	Group			Time			Group × Time		
	*F*	*p*	*η* ^2^	*F*	*p*	*η* ^2^	*F*	*p*	*η* ^2^
SBP	2.983	0.093	0.077	0.221	0.641	0.006	0.251	0.619	0.007
DBP	0.695	0.410	0.018	0.145	0.705	0.004	0.209	0.650	0.005
Body fat %	0.657	0.423	0.017	0.172	0.681	0.005	2.848	0.100	0.071
Fat mass	0.311	0.580	0.008	0.014	0.907	0.000	3.565	0.067	0.088
Lean mass	0.002	0.969	0.000	0.066	0.798	0.002	0.012	0.912	0.012
BMI	0.253	0.618	0.007	0.045	0.833	0.001	3.402	0.073	0.082

Notes: SBP = systolic blood pressure; DBP = diastolic blood pressure; Body fat % = body fat percentage; BMI = body mass index. Effect size (*η*^2^) is interpreted as small (*η*^2^ = 0.01), medium (*η*^2^ = 0.06), and large (*η*^2^ = 0.14).

**Table 4:** Descriptive and inferential statistics for daily average step count for the Control and PB groups for all weeks of the study. Descriptive data are presented as: mean (standard deviation).

Time	Control	PB	*df*	*t*	*p*	*g*
Baseline	11,251.49 (2576.85)	12,601.71 (4107.21)	36	1.23	0.23	0.39
Week 2	10,842.34 (2691.97)	12,483.90 (3737.00)	35	1.53	0.14	0.49
Week 3	10,936.08 (3576.59)	11,540.02 (2533.28)	29	0.53	0.60	0.19
Week 4	11,070.78 (3992.34)	10,907.17 (2930.67)	22	0.11	0.91	0.05
Week 5	12,802.25 (4258.19)	10,374.00 (5951.74)	17	0.98	0.34	0.44
Week 6	12,799.63 (2737.32)	9651.28 (3275.27)	15	2.08	0.06	0.97
Week 7	11,994.06 (1762.88)	10,860.24 (3330.22)	10	0.69	0.51	0.37
Week 8	13,182.00 (2886.75)	11,411.02 (4297.25)	6	0.68	0.52	0.42

Note: PB = Peanut Butter group. Effect size is presented as Hedge’s correction (*g*) and interpreted as small (*g* = 0.2), medium (*g* = 0.5), and large (*g* = 0.8).

## Data Availability

Documents with statistical analysis output are available at the following link to the Open Science Framework repository: https://osf.io/j3bx4/ (accessed on 11 March 2024).

## References

[1] Alper C.M., Mattes R.D. (2002). Effects of chronic peanut consumption on energy balance and hedonics. Int. J. Obes. Relat. Metab. Disord..

[2] Bhat E.A., Sajjad N., Manzoor I., Rasool A. (2019). Bioactive compounds in peanuts and banana. Anal. Biochem..

[3] Arya S.S., Salve A.R., Chauhan S. (2016). Peanuts as functional food: A review. J. Food Sci. Technol..

[4] Isanga J., Zhang G.N. (2007). Biologically active components and nutraceuticals in peanuts and related products. Food Rev. Int..

[5] Kris-Etherton P.M., Pearson T.A., Wan Y., Hargrove R.L., Moriarty K., Fishell V., Etherton T.D. (1999). High-monounsaturated fatty acid diets lower both plasma cholesterol and triacylglycerol concentrations. Am. J. Clin. Nutr..

[6] Higgs J. (2005). The potential role of peanuts in the prevention of obesity. Nutr. Food Sci..

[7] Ghadimi Nouran M., Kimiagar M., Abadi A., Mirzazadeh M., Harrison G. (2010). Peanut consumption and cardiovascular risk. Public Health Nutr..

[8] Petersen K.S., Murphy J., Whitbread J., Clifton P.M., Keogh J.B. (2022). The Effect of a Peanut-Enriched Weight Loss Diet Compared to a Low-Fat Weight Loss Diet on Body Weight, Blood Pressure, and Glycemic Control: A Randomized Controlled Trial. Nutrients.

[9] Jones J.B., Provost M., Keaver L., Breen C., Ludy M.J., Mattes R.D. (2014). A randomized trial on the effects of flavorings on the health benefits of daily peanut consumption. Am. J. Clin. Nutr..

[10] Sithole T.R., Ma Y., Qin Z., Wang X., Liu H. (2025). A comparative analysis of the nutritional and physicochemical properties of peanut butter paste produced from raw, roasted, and boiled peanuts. Front. Food Sci. Technol..

[11] Kales S.N., Soteriades E.S., Christophi C.A., Christiani D.C. (2007). Emergency duties and deaths from heart disease among firefighters in the United States. N. Engl. J. Med..

[12] Fahs C.A., Smith D.L., Horn G.P., Agiovlasitis S., Rossow L.M., Echols G., Heffernan K.S., Fernhall B. (2009). Impact of excess body weight on arterial structure, function, and blood pressure in firefighters. Am. J. Cardiol..

[13] Bode E.D., Mathias K.C., Stewart D.F., Moffatt S.M., Jack K., Smith D.L. (2021). Cardiovascular Disease Risk Factors by BMI and Age in United States Firefighters. Obesity.

[14] Superko H.R., Momary K.M., Pendyala L.K., Williams P.T., Frohwein S., Garrett B.C., Skrifvars C., Gadesam R., King S.B., Rolader S. (2011). Firefighters, heart disease, and aspects of insulin resistance: The FEMA Firefighter Heart Disease Prevention study. J. Occup. Environ. Med..

[15] Soteriades E.S., Smith D.L., Tsismenakis A.J., Baur D.M., Kales S.N. (2011). Cardiovascular disease in US firefighters: A systematic review. Cardiol. Rev..

[16] Cappuccio F.P., D’Elia L., Strazzullo P., Miller M.A. (2010). Sleep duration and all-cause mortality: A systematic review and meta-analysis of prospective studies. Sleep.

[17] Angleman A.J., Van Hasselt V.B., Schuhmann B.B. (2022). Relationship Between Posttraumatic Stress Symptoms and Cardiovascular Disease Risk in Firefighters. Behav. Modif..

[18] Jeung D.Y., Hyun D.S., Kim I., Chang S.J. (2022). Effects of Emergency Duties on Cardiovascular Diseases in Firefighters: A 13-Year Retrospective Cohort Study. J. Occup. Environ. Med..

[19] Chobanian A.V., Bakris G.L., Black H.R., Cushman W.C., Green L.A., Izzo J.L., Jones D.W., Materson B.J., Oparil S., Wright J.T. (2003). The Seventh Report of the Joint National Committee on Prevention Detection Evaluation Treatment of High Blood Pressure: The JNC7 report. JAMA.

[20] Kales S.N., Tsismenakis A.J., Zhang C., Soteriades E.S. (2009). Blood pressure in firefighters, police officers, and other emergency responders. Am. J. Hypertens..

[21] Sawicka K., Szczyrek M., Jastrzebska I., Prasal M., Zwolak A., Daniluk J. (2011). Hypertension—The silent killer. J. Pre-Clin. Res..

[22] Soteriades E.S., Kales S.N., Liarokapis D., Christiani D.C. (2003). Prospective surveillance of hypertension in firefighters. J. Clin. Hypertens..

[23] Crea F. (2022). Update on a silent killer: Arterial hypertension. Eur. Heart J..

[24] Noh J., Lee C.J., Hyun D.S., Kim W., Kim M.J., Park K.S., Koh S., Chang S.J., Kim C., Park S. (2020). Blood pressure and the risk of major adverse cardiovascular events among firefighters. J. Hypertens..

[25] Dobson M., Choi B., Schnall P.L., Wigger E., Garcia-Rivas J., Israel L., Baker D.B. (2013). Exploring occupational and health behavioral causes of firefighter obesity: A qualitative study. Am. J. Ind. Med..

[26] Tsismenakis A.J., Christophi C.A., Burress J.W., Kinney A.M., Kim M., Kales S.N. (2009). The obesity epidemic and future emergency responders. Obesity.

[27] Kay B.F., Lund M.M., Taylor P.N., Herbold N.H. (2001). Assessment of firefighters’ cardiovascular disease-related knowledge and behaviors. J. Am. Diet. Assoc..

[28] Glueck C.J., Kelley W., Wang P., Gartside P.S., Black D., Tracy T. (1996). Risk factors for coronary heart disease among firefighters in Cincinnati. Am. J. Ind. Med..

[29] Kirkmeyer S.V., Mattes R.D. (2000). Effects of food attributes on hunger and food intake. Int. J. Obes. Relat. Metab. Disord..

[30] van Marken Lichtenbelt W.D., Mensink R.P., Westerterp K.R. (1997). The effect of fat composition of the diet on energy metabolism. Z. Ernährungswissenschaft.

[31] Blanca M.J., Alarcón R., Arnau J., Bono R., Bendayan R. (2017). Non-normal data: Is ANOVA still a valid option?. Psicothema.

[32] Cohen J. (1988). Statistical Power Analysis for the Behavioral Sciences.

[33] Baek S.H., Park J.J., Seo D.I., Song W., Lee C.G., Lee H.J., Ahn Y.S., Kang H.J. (2018). Systematic review of varied exercise programs on body composition and physical fitness for firefighters. Asian J. Kinesiol..

[34] Yang J., Farioli A., Korre M., Kales S.N. (2015). Dietary Preferences and Nutritional Information Needs Among Career Firefighters in the United States. Glob. Adv. Health Med..

[35] Joe M.J., Hatsu I.E., Tefft A., Mok S., Adetona O. (2022). Dietary Behavior and Diet Interventions among Structural Firefighters: A Narrative Review. Nutrients.

[36] Ras R.T., Zock P.L., Zebregs Y.E., Johnston N.R., Webb D.J., Draijer R. (2013). Effect of polyphenol-rich grape seed extract on ambulatory blood pressure in subjects with pre- and stage I hypertension. Br. J. Nutr..

[37] Tjelle T.E., Holtung L., Bøhn S.K., Aaby K., Thoresen M., Wiik S.Å., Paur I., Karlsen A.S., Retterstøl K., Iversen P.O. (2015). Polyphenol-rich juices reduce blood pressure measures in a randomised controlled trial in high normal and hypertensive volunteers. Br. J. Nutr..

[38] McKiernan F., Lokko P., Kuevi A., Sales R.L., Costa N.M., Bressan J., Alfenas R.C., Mattes R.D. (2010). Effects of peanut processing on body weight and fasting plasma lipids. Br. J. Nutr..

[39] Mohammadifard N., Salehi-Abargouei A., Salas-Salvadó J., Guasch-Ferré M., Humphries K., Sarrafzadegan N. (2015). The effect of tree nut, peanut, and soy nut consumption on blood pressure: A systematic review and meta-analysis of randomized controlled clinical trials. Am. J. Clin. Nutr..

[40] Nagpal R., Patoine C., Singar S., Lobene A., Fenner S., Houser L. Effect of Peanut Butter on Gut and Metabolic Health. ClinicalTrials.gov Identifier: NCT06916936. NCT06916936.

[41] Wattanapisit A., Thanamee S. (2013). Evidence behind 10,000 steps walking. J. Health Res..

